# Neurovascular coupling impairment as a mechanism for cognitive deficits in COVID-19

**DOI:** 10.1093/braincomms/fcae080

**Published:** 2024-03-07

**Authors:** Cameron D Owens, Camila Bonin Pinto, Sam Detwiler, Lauren Olay, Ana Clara da C Pinaffi-Langley, Peter Mukli, Anna Peterfi, Zsofia Szarvas, Judith A James, Veronica Galvan, Stefano Tarantini, Anna Csiszar, Zoltan Ungvari, Angelia C Kirkpatrick, Calin I Prodan, Andriy Yabluchanskiy

**Affiliations:** Oklahoma Center for Geroscience and Healthy Brain Aging, University of Oklahoma Health Sciences Center, Oklahoma City, OK 73117, USA; Vascular Cognitive Impairment, Neurodegeneration and Healthy Brain Aging Program, Department of Neurosurgery, University of Oklahoma Health Sciences Center, Oklahoma City, OK 73104, USA; Oklahoma Center for Geroscience and Healthy Brain Aging, University of Oklahoma Health Sciences Center, Oklahoma City, OK 73117, USA; Vascular Cognitive Impairment, Neurodegeneration and Healthy Brain Aging Program, Department of Neurosurgery, University of Oklahoma Health Sciences Center, Oklahoma City, OK 73104, USA; Oklahoma Center for Geroscience and Healthy Brain Aging, University of Oklahoma Health Sciences Center, Oklahoma City, OK 73117, USA; Oklahoma Center for Geroscience and Healthy Brain Aging, University of Oklahoma Health Sciences Center, Oklahoma City, OK 73117, USA; Oklahoma Center for Geroscience and Healthy Brain Aging, University of Oklahoma Health Sciences Center, Oklahoma City, OK 73117, USA; Oklahoma Center for Geroscience and Healthy Brain Aging, University of Oklahoma Health Sciences Center, Oklahoma City, OK 73117, USA; Vascular Cognitive Impairment, Neurodegeneration and Healthy Brain Aging Program, Department of Neurosurgery, University of Oklahoma Health Sciences Center, Oklahoma City, OK 73104, USA; International Training Program in Geroscience, Doctoral School of Basic and Translational Medicine/Departments of Public Health, Translational Medicine and Physiology, Semmelweis University, Budapest, 1089, Hungary; Oklahoma Center for Geroscience and Healthy Brain Aging, University of Oklahoma Health Sciences Center, Oklahoma City, OK 73117, USA; Vascular Cognitive Impairment, Neurodegeneration and Healthy Brain Aging Program, Department of Neurosurgery, University of Oklahoma Health Sciences Center, Oklahoma City, OK 73104, USA; International Training Program in Geroscience, Doctoral School of Basic and Translational Medicine/Departments of Public Health, Translational Medicine and Physiology, Semmelweis University, Budapest, 1089, Hungary; Oklahoma Center for Geroscience and Healthy Brain Aging, University of Oklahoma Health Sciences Center, Oklahoma City, OK 73117, USA; Vascular Cognitive Impairment, Neurodegeneration and Healthy Brain Aging Program, Department of Neurosurgery, University of Oklahoma Health Sciences Center, Oklahoma City, OK 73104, USA; International Training Program in Geroscience, Doctoral School of Basic and Translational Medicine/Departments of Public Health, Translational Medicine and Physiology, Semmelweis University, Budapest, 1089, Hungary; Oklahoma Center for Geroscience and Healthy Brain Aging, University of Oklahoma Health Sciences Center, Oklahoma City, OK 73117, USA; Arthritis & Clinical Immunology Research Program, Oklahoma Medical Research Foundation, Oklahoma City, OK 73104, USA; Department of Internal Medicine, University of Oklahoma Health Sciences Center, Oklahoma City, OK 73104, USA; Department of Pathology, University of Oklahoma Health Sciences Center, Oklahoma City, OK 73104, USA; Oklahoma Center for Geroscience and Healthy Brain Aging, University of Oklahoma Health Sciences Center, Oklahoma City, OK 73117, USA; Department of Biochemistry and Molecular Biology, University of Oklahoma Health Sciences Center, Oklahoma City, OK 73104, USA; Veterans Affairs Medical Center, Oklahoma City, OK 73104, USA; Oklahoma Center for Geroscience and Healthy Brain Aging, University of Oklahoma Health Sciences Center, Oklahoma City, OK 73117, USA; Vascular Cognitive Impairment, Neurodegeneration and Healthy Brain Aging Program, Department of Neurosurgery, University of Oklahoma Health Sciences Center, Oklahoma City, OK 73104, USA; International Training Program in Geroscience, Doctoral School of Basic and Translational Medicine/Departments of Public Health, Translational Medicine and Physiology, Semmelweis University, Budapest, 1089, Hungary; The Peggy and Charles Stephenson Cancer Center, University of Oklahoma Health Sciences Center, Oklahoma City, OK 73104, USA; Department of Health Promotion Sciences, College of Public Health, University of Oklahoma Health Sciences Center, Oklahoma City, OK 73104, USA; Oklahoma Center for Geroscience and Healthy Brain Aging, University of Oklahoma Health Sciences Center, Oklahoma City, OK 73117, USA; Vascular Cognitive Impairment, Neurodegeneration and Healthy Brain Aging Program, Department of Neurosurgery, University of Oklahoma Health Sciences Center, Oklahoma City, OK 73104, USA; International Training Program in Geroscience, Doctoral School of Basic and Translational Medicine/Departments of Public Health, Translational Medicine and Physiology, Semmelweis University, Budapest, 1089, Hungary; Oklahoma Center for Geroscience and Healthy Brain Aging, University of Oklahoma Health Sciences Center, Oklahoma City, OK 73117, USA; Vascular Cognitive Impairment, Neurodegeneration and Healthy Brain Aging Program, Department of Neurosurgery, University of Oklahoma Health Sciences Center, Oklahoma City, OK 73104, USA; International Training Program in Geroscience, Doctoral School of Basic and Translational Medicine/Departments of Public Health, Translational Medicine and Physiology, Semmelweis University, Budapest, 1089, Hungary; Department of Health Promotion Sciences, College of Public Health, University of Oklahoma Health Sciences Center, Oklahoma City, OK 73104, USA; Veterans Affairs Medical Center, Oklahoma City, OK 73104, USA; Cardiovascular Section, Department of Medicine, University of Oklahoma Health Sciences Center, Oklahoma City, OK 73117, USA; Veterans Affairs Medical Center, Oklahoma City, OK 73104, USA; Department of Neurology, University of Oklahoma Health Sciences Center, Oklahoma City, OK 73104, USA; Oklahoma Center for Geroscience and Healthy Brain Aging, University of Oklahoma Health Sciences Center, Oklahoma City, OK 73117, USA; Vascular Cognitive Impairment, Neurodegeneration and Healthy Brain Aging Program, Department of Neurosurgery, University of Oklahoma Health Sciences Center, Oklahoma City, OK 73104, USA; International Training Program in Geroscience, Doctoral School of Basic and Translational Medicine/Departments of Public Health, Translational Medicine and Physiology, Semmelweis University, Budapest, 1089, Hungary; Department of Health Promotion Sciences, College of Public Health, University of Oklahoma Health Sciences Center, Oklahoma City, OK 73104, USA

**Keywords:** cognitive impairment, endothelium, SARS-CoV-2, neurovascular uncoupling, oxidative stress

## Abstract

Components that comprise our brain parenchymal and cerebrovascular structures provide a homeostatic environment for proper neuronal function to ensure normal cognition. Cerebral insults (e.g. ischaemia, microbleeds and infection) alter cellular structures and physiologic processes within the neurovascular unit and contribute to cognitive dysfunction. COVID-19 has posed significant complications during acute and convalescent stages in multiple organ systems, including the brain. Cognitive impairment is a prevalent complication in COVID-19 patients, irrespective of severity of acute SARS-CoV-2 infection. Moreover, overwhelming evidence from in vitro, preclinical and clinical studies has reported SARS-CoV-2-induced pathologies in components of the neurovascular unit that are associated with cognitive impairment. Neurovascular unit disruption alters the neurovascular coupling response, a critical mechanism that regulates cerebromicrovascular blood flow to meet the energetic demands of locally active neurons. Normal cognitive processing is achieved through the neurovascular coupling response and involves the coordinated action of brain parenchymal cells (i.e. neurons and glia) and cerebrovascular cell types (i.e. endothelia, smooth muscle cells and pericytes). However, current work on COVID-19-induced cognitive impairment has yet to investigate disruption of neurovascular coupling as a causal factor. Hence, in this review, we aim to describe SARS-CoV-2's effects on the neurovascular unit and how they can impact neurovascular coupling and contribute to cognitive decline in acute and convalescent stages of the disease. Additionally, we explore potential therapeutic interventions to mitigate COVID-19-induced cognitive impairment. Given the great impact of cognitive impairment associated with COVID-19 on both individuals and public health, the necessity for a coordinated effort from fundamental scientific research to clinical application becomes imperative. This integrated endeavour is crucial for mitigating the cognitive deficits induced by COVID-19 and its subsequent burden in this especially vulnerable population.

## Teaser

Owens *et al*. determined SARS-CoV-2 as a driver of neurovascular unit disruption and cognitive impairment. In this review, they were the first to propose neurovascular uncoupling as a mechanism for COVID-19-induced cognitive deterioration.

## Graphical Abstract

**Graphical Abstract:**
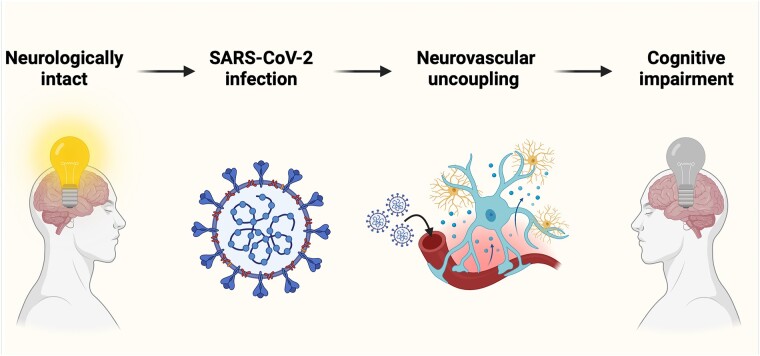

## Introduction

As of February 2024, COVID-19 has affected over 770 million individuals with 7 million COVID-19 attributed deaths worldwide.^1^ Acute SARS-CoV-2 infection typically proceeds as a mild to moderate infection with common symptoms of fever, fatigue and dry cough.^2^ However, in older patients with vascular comorbidities [i.e. type 2 diabetes (T2D), hypertension], SARS-CoV-2 infection pathogenicity rises, involving multi-organ dysfunction and increased risk of death.^3-5^ The brain is a critical target organ in acute and recovered COVID-19 patients^6^ experiencing long-term symptoms (i.e. Long-COVID).^7^ Clinical evidence has indicated that up to 70% of patients enduring persistent COVID-19 symptoms after recovery from initial infection report cognitive deficits.^8^ Moreover, COVID-19 related cognitive sequela are more prominent in older adults with underlying complications,^9^ making prompt investigation into mechanisms of COVID-19-induced cognitive impairment of the utmost importance for this vulnerable patient population. However, pathophysiological mechanisms have yet to be uncovered for cognitive dysfunction preceded by COVID-19.

Neurovascular coupling (NVC) is a critical mechanism to ensure normal cognitive processing and involves the coordinated action of brain parenchymal (i.e. neurons and glia) and vascular cells [i.e. endothelia, smooth muscle (SMC), pericytes] that comprise the neurovascular unit (NVU).^10^ NVC is responsible for temporally and spatially precise adjustment of cerebromicrovascular blood flow to meet the energetic and nutritive needs of active neurons.^11^ Disruption of the NVU is documented in preclinical models and contributes to impaired NVC and resulting cognitive dysfunction.^12^ Moreover, clinical studies have replicated these findings, showing impaired NVC responses in aging^13-15^ and age-related disorders [i.e. T2D,^16^ peripheral artery disease,^17-19^ cerebral small vessel disease (CSVD)^20^ and dementia].^21,22^

The endothelium is an essential player in the NVC response. In direct communication with glial and neuronal cell types on the parenchymal side, and contractile pericytes and SMCs superficial to the vascular intima, the endothelium is in an ideal position to receive neuronal and glial signalling to mediate the vasodilatory response.^10^ Impairment of the endothelium in common age-related conditions disrupts the NVC response, contributing to cognitive dysfunction.^23^ In addition, clinical studies showed that widespread macro- and microvascular dysfunction strongly associated with NVC impairment and cognitive decline.^17,24^ SARS-CoV-2 has effects on all cells within the NVU; however, permissibility of cell types is variable, with the endothelium largely deemed as the most permissible, and likely involved in mediating the neuropathologic consequences associated throughout the NVU.^25^ Systemic vascular dysfunction is prevalent in acute and convalescent COVID-19.^26^ Moreover, evidence suggests that SARS-CoV-2 may have specific neuroanatomical predilection of CSVD pathology (i.e. cerebral microbleeds) that is associated with cognitive impairment.^6^ Hence, the SARS-CoV-2 virus is at the forefront of NVU disruption, but NVC impairment has yet to be investigated as a mechanism for COVID-19-induced cognitive sequela.

This review aims to (i) detail COVID-19 as a factor that contributes to, and exacerbates, cognitive impairment, (ii) review components of the NVC response and COVID-19's role in NVU and NVC disruption and (iii) provide evidence of preventative and therapeutic treatment to mitigate COVID-19-induced cognitive impairment through rescue of NVC. Neurovascular dysfunction is one of the most prominent, as well as a preceding feature, in cognitive decline and progression to dementia.^27^ Hence, we aim to unravel the COVID-19-induced effects on NVC and cognition to pave the way for bench to bedside research and clinical therapeutic intervention.

## Cognitive impairment in COVID-19

COVID-19-induced cognitive impairment is highly prevalent, with nearly one in every four patients presenting with cognitive deficits up to 1 year following infection.^28-30^ Inability to critically think and remember past events is the one of leading causes of disability in older adults with ∼25% of individuals over 65 presenting with cognitive dysfunction in the United States.^31^ Moreover, this impairment worsens in pathological neurocognitive disorders such as mild cognitive impairment (MCI) and dementia. A looming question remains: how long do the effects of COVID-19 on cognition last? After 3 years post-pandemic, we have yet to reach this answer, as the risk horizon (i.e. no greater risk between COVID-19 and control) and time to equal incidence (i.e. equal cumulative incidence between groups) have not arrived.^32^ Hence, it is imperative to investigate mechanisms that contribute to COVID-19-induced cognitive impairment and develop therapeutic interventions to mitigate cognitive decline following recovery from acute SARS-CoV-2 infection. Here, we highlight information from systematic reviews and meta-analyses (Table 1) assessing COVID-19-induced cognitive impairment.

**Table 1:** Meta-analysis evidence of long-term post-COVID-19 cognitive sequela

Year	Area of focus	Evidence
2023	Executive function impairment in the post-COVID population compared to healthy controls	Velichkovsky *et al*. evaluated six studies involving 9411 participants using fluid cognitive tests and found that post-COVID-19 individuals demonstrated a reduction in executive functioning, including inhibition and set shifting, when compared with healthy controls [Standardized Mean Difference −0.35, 95% CI (−0.59, −0.11), *P* < 0.05].
2022	Prevalence of neurologic sequelae of COVID-19 infection persisting for one year following diagnosis	Zeng *et al*. evaluated 68 studies and 738 430 participants and found that nearly 20% of patients recovering from COVID-19 experienced general cognitive impairment [19.7%, 95% CI (8.8–33.4%)] while ∼18% [17.5%, 95% CI (8.6–29.6%)] suffered from memory impairment in the post-COVID phase. Furthermore, cognitive impairment was found to be a persistent side effect of COVID-19 infection with ∼22% of individuals [22.2%, 95% CI (19.6–25.0%)] continuing to experience cognitive deficits 6–12 months following infection.
	General cognitive impairment in the post-COVID population compared to healthy controls	Crivelli *et al*. evaluated 27 studies involving 2049 participants using primarily the Montreal Cognitive Assessment and found that individuals recovering from COVID-19 infection demonstrated a significant decline in general cognitive functioning at 6-month follow-up compared to healthy controls [−0.94, 95% CI (−1.59, −0.29); *P* = 0.0049]. Additionally, post-COVID-19 individuals demonstrated persistent decreased performance in executive functioning, memory, and attention when compared to healthy individuals.
	Long-term neurologic impact of COVID-19 infection in recovered adult patients	Premraj *et al*. evaluated 18 studies involving 10 530 participants and found that nearly 30% of individuals recovering from COVID-19 experienced memory impairment [28.44%, 95% CI (21.52–35.35%)], 21% experienced attention deficits [21.84%, 95% CI (7.30–36.38%)], and 32% reported a subjective sense of ‘brain fog’ [32.17%, 95% CI (10.31–54.03%)] presenting within 3 months following infection and persisting as a long-term sequalae (6 months or more) of the disease.
2021	Prevalence of persistent subjective and objective cognitive impairment in individuals recovered from COVID-19 infection	Badenoch *et al*. evaluated 51 studies involving 18 917 and found that ∼20% of participants [20.2%, 95% CI (10.3–35.7%)] experienced cognitive impairment assessed by objective measurements such as Montreal Cognitive Assessment. It was determined that 15% of participants [15.3%, 95% CI (8.9–25%)] experienced subjective cognitive impairment assessed using self-reporting of memory impairment, concentration impairment, or ‘brain fog’.

Multiple cognitive domains are impaired following SARS-CoV-2 infection. Executive function, controlled largely by the prefrontal cortex and critically involved in higher-level thinking and decision making, is one of these domains.^33^ Specific deficits of executive function include planning, abstraction, behavioural control and orientation.^28,34,35^ Other functions of the prefrontal cortex, including attention and working memory, are also commonly impaired after recovery from COVID-19.^28,34,36^ Visuospatial and episodic memory impairment are a consistent finding following SARS-CoV-2 infection.^28,34^ In recovered COVID-19 patients, visuospatial abnormalities are associated with increased burden of white matter hyperintensities,^34^ a common marker of ischaemic damage to the brain parenchyma due to CSVD.^37^ These findings are critical in understanding underlying aetiologies for COVID-19 induced cognitive impairment, as COVID-19 has a specific neuroanatomical predilection of CSVD and associated with cognitive dysfunction.^6^ Episodic memory, an area controlled largely by the medial temporal lobe and hippocampus, is also as an impaired cognitive domain following SARS-CoV-2 infection.^34^ Importantly, as these symptoms, along with COVID-19 neuropathology, have similar features to Alzheimer's disease,^38,39^ there is an urgent need to unravel a mechanism that contributes to this detrimental progression of cognitive impairment.

The impact of illness severity on cognitive impairment has been a significant focus of investigation. In multiple systematic reviews encompassing over 25 separate studies of COVID-19-induced cognitive dysfunction, severity of acute infection was not shown to chiefly contribute to onset of cognitive impairment^28,40^ Vascular comorbidities (i.e. T2D, hypertension and dyslipidemia) are the most common modifiable risk factors of dementia progression.^41^ Cognitive impairment post-COVID-19 is associated with concomitant vascular comorbidities; however, patients with a history of COVID-19 had an 18% increase in cognitive impairment prevalence compared with controls, even after adjusting for vascular comorbidities.^33^ Hence, COVID-19 is likely an independent factor in cognitive impairment development. Given COVID-19's classification as a vascular disease primarily affecting endothelial function,^42^ its recognition as an additional risk factor of cognitive impairment, particularly when combined with established vascular comorbidities, represents a defining moment in our ongoing understanding of long-term cognitive sequela.

In addition to the impact of SARS-CoV-2 on cognitive health, extrinsic factors from the COVID-19 pandemic and lock down procedures posed challenges that may have affected cognition. Prolonged indoor settings and limited social interaction have negative impacts on brain health and increases the risk of depression.^43^ During the peak of the COVID-19 pandemic there was an elevated incidence of depression.^44^ Depression itself an independent risk factor for development of cognitive impairment.^45^ Social isolation at the onset of the pandemic altered sleep/wake cycles, which is a predictor of depression^46^ and affects cardiovascular function^47,48^ and functional cognitive processes.^49,50^ Physical activity levels of individuals decreased world-wide during the height of the pandemic.^51^ Moderate physical exercise beginning in midlife has been showing to significantly reduce risk of cognitive decline.^52^ Hence, physical exercise could potentially aid in alleviating long-COVID brain fog in middle-aged to older adults.

## Neurovascular coupling mechanism and COVID-19-induced cell-specific impairment

**Figure 1:**
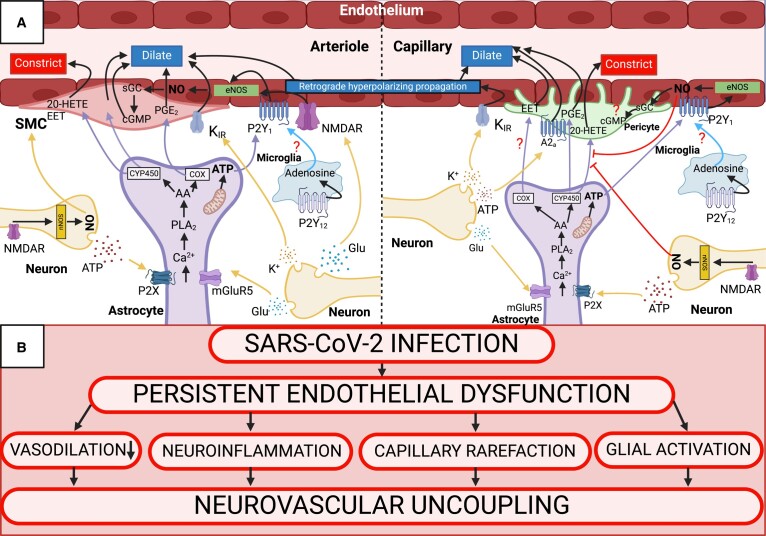
**NVC response and SARS-CoV-2-mediated neurovascular disruption.** (**A**) NVC response at the arteriolar level (left) and capillary level (right). Arrows with question marks indicate hypothesized pathways yet to be uncovered. Sharp arrows indicate cellular signaling within the NVU. Flathead arrows indicate inhibitory action on NVU pathways. (**B**) Hypothesized SARS-CoV-2-mediated neurovascular uncoupling. SARS-CoV-2 known action of persistent endothelial dysfunction is hypothesized as a causal factor that underlies vasodilatory impairment, neuroinflammation, capillary rarefaction, and glial activation–all known factors that contribute to neurovascular uncoupling. Created with BioRender.com. 20-HETE = 20-hydroxyeicosatetraenoic acid; cGMP = cyclic guanosine monophosphate; sGC = soluble guanylate cyclase; nNOS = neuronal nitric oxide synthase; PGE_2_ = prostaglandin E_2_; P2X = purinergic ligand-gated ion channel; AA = arachidonic acid; CYP450 = cytochrome P450; P2Y_1_, P2Y_12_ = purinergic G protein-coupled receptors.

Cognitive function is intrinsically linked to the homeostatic mechanism of NVC. In its most simplistic form, increased local neuronal activity initiates the process of NVC, or functional hyperaemia, by eliciting spatially and temporally precise cerebromicrovascular vasodilation and increased blood flow through direct or indirect mechanisms. Due to a lack of neuronal energy reserves, this tight coupling mechanism is essential for efficient delivery of oxygen and glucose to meet local neuronal demand.^11^ Moreover, neurotoxic metabolites (e.g. CO_2_ and amyloid-β peptide) are cleared through NVC-mediated increase in blood flow, thereby creating a homeostatic microenvironment to ensure proper neuronal function.^10,53^ Acute disease and progression of long-term symptoms in COVID-19 are detrimental to brain function. As COVID-19 has been deemed an endothelial disease^42,54-56^ with prevalent cerebral small and large vessel complications arising from endothelial impairment, we describe COVID-19 mechanism of infection in the context of the cerebrovascular endothelium. Moreover, specific pathways within the NVU that contribute to NVC, along with hypothesized SARS-CoV-2-mediated effects on the NVC response, are represented in Fig. 1. Here, we will cover cell-specific interactions within the NVC response, and *in vitro*, preclinical, and clinical evidence of SARS-CoV-2 cell-specific impairment.

### Mechanism of infection

Originally deemed as a respiratory epithelial infection,^57^ the systemic vasculature, with specificity to the endothelium, quickly came to the forefront of COVID-19 induced multi-organ impairment.^42^ The impact on the brain is of specific interest as cognitive dysfunction and cerebral macro- and microvascular pathologies are prevalent in acute and Long-COVID patients.^6^ Moreover, the endothelium is heterogeneous in structure and function at different areas the body,^58^ and SARS-CoV-2 utilizes region specific cellular characteristics of the cerebrovascular endothelium to elicit brain pathology.

At the onset of cerebrovascular endothelial infection, SARS-CoV-2 spike (S1) glycoprotein attaches to the angiotensin converting enzyme 2 (ACE2) receptor. ACE2 is a key contributor in the renin-angiotensin pathway, cleaving the leucyl residue from angiotensin (Ang) I and AngII, producing Ang_1–9_ and Ang_1–7_, respectively.^59^ Ang_1–7_ acting on the Mas receptor (ACE2-Ang_1–7_-Max axis) is neuroprotective,^60^ while ACE-Ang II-Ang II receptor type 1 (AT1R) binding contributes to endothelial activation [i.e. elevations in pro-inflammatory cytokines, reactive oxygen species (ROS), prothrombotic factors] and a persistent thromboinflammatory state.^19,61^

Following S1 binding, upregulation of proteases ADAM17 and TMPRSS2 (a disintegrin and metallopeptidase domain 17 and transmembrane serine protease 2) lead to ACE2 shedding,^62^ resulting in uninhibited binding of AngII to AT1R and loss of the neuroprotective ACE2-Ang_1–7_-Mas axis. The ensuing endothelial activation and loss of ACE2 lead to upregulation of leukocyte adhesion molecule expression^19,59^ and bradykinin binding to B2 receptors, all contributing to leukocyte transmigration and blood brain barrier disruption (BBB). Moreover, this state of endothelial activation triggers platelet activation, resulting in a fibrin clot and tissue plasminogen activator (tPA)-mediated plasmin formation with concomitant bradykinin production, further disrupting BBB integrity.^18,19,63,64^

After acute infection, there is strong clinical evidence of long term endothelial dysfunction,^55^ but the mechanism has not been uncovered. However, highly plausible mechanisms have been hypothesized with the theory of chronically elevated mitochondrial driven ROS generation and suppression of NO production as a mechanism for long-term endothelial dysfunction.^65^ NO is widely known for its roles as a gasotransmitter responsible for vasodilatory response. This molecule is also critically involved in maintaining the quiescent state of the endothelium,^66^ and when production is disrupted, is causally related to vascular pathologic consequences, including impairment of NVC and cognitive decline.^27,67^

### Neurons

At initiation of NVC, neurons release glutamate, and activity at glutaminergic post-synaptic receptors, N-methyl-D-aspartate (NMDA) and α-amino-3-hydroxy-5-methyl-4-isoxazol propionic acid (AMPA), lead to elevations in intracellular calcium ion (Ca^2+^) concentration that activate Ca^2+^ dependent enzymes [i.e. neuronal nitric oxide synthase (nNOS), cyclooxygenase 2 (COX-2)] and produce vasodilatory molecules [i.e. nitric oxide (NO), prostanoids].^68,69^ Moreover, glutamate can act at metabotropic glutamate receptors (mGluR) and ionotropic receptors (NMDAR) on glial and vascular cells, respectively, contributing to the NVC response.^27^ Elevations in extracellular potassium ion (K^+^) concentration, due to excitatory neuronal activity, may also facilitate the vascular response,^70^ and neuronal release of adenosine and adenosine triphosphate (ATP), potent vasodilators, have been reported to act on purinergic receptors and elicit increases in local cerebromicrovascular blood flow.^10^

There are many possibilities regarding how SARS-CoV-2 may disrupt normal neuronal function.^71^ The strongest possibility is COVID-19 induced cerebrovascular dysfunction contributing to BBB disruption, clotting cascade abnormalities and endothelial activation (i.e. elevated cytokine secretion and ROS, all of which are known to disrupt neuronal function and will be discussed in the *Endothelia* and *Mural cell* sections. Other mechanisms are also plausible and most likely work in tandem. In a mouse model of SARS-CoV-2 infection that was bioengineered to restrict SARS-CoV-2 infection to the respiratory system, the virus was cleared in a week and did not enter the brain. However, the infection-induced immune response resulted in elevated cerebral spinal fluid (CSF) cytokines and impaired hippocampal neurogenesis, most likely related to increased microglial reactivity seen following infection.^72^ Moreover, these findings have been replicated in other preclinical models and decreased hippocampal neurons and global brain atrophy have been seen at autopsy of COVID-19 patients.^73,74^ These findings highlight the immune-glial (discussed in *Astrocyte* and *Microglial* sections) interaction and the effect of respiratory specific infection on neuronal cells without spread to other organ systems. Autoimmunity may also contribute to SARS-CoV-2 induced neuronal dysfunction as COVID-19 patient CSF revealed auto-antibodies to neuronal and cerebrovascular cell types.^75,76^ Reactivation of latent pathogens (i.e. Epstein-Barr virus [EBV]) is another possibility^77^ for neuronal damage and has been postulated as one of the mechanisms of Long-COVID.^55^ EBV reactivation is typically associated with immunocompromised patients. However, in COVID-19, the physiological stress induced by acute infection has the potential to reactivate latent viruses. This, in turn, may contribute to the neurological sequela^78^ observed in Long-COVID, possibly through mechanisms involving direct neuronal infection and/or BBB disruption.^79^ Clinical evidence suggests that SARS-CoV-2 direct neuronal infection is rare and does not account for the neurological sequela associated with COVID-19.^71^ Autopsy and CSF studies from COVID-19 patients with neurological symptoms show no detection of SARS-CoV-2 in the brain or CSF.^75,80-84^ In addition, *in vitro* studies of SARS-CoV-2 original, Delta, and Omicron variants did not infect neurons or astrocytes, but microglia were highly permissive.^85^ The non-permissive nature of neurons to infection through the endocytic ACE2 pathway is due to low or absent ACE2 receptors. In contrast, others have reported SARS-CoV-2 in the post-mortem brain from COVID-19 patients.^86^ This may occur through alternate pathways of neuronal infection, such as tunnelling nanotubes, and provide a route for SARS-CoV-2 spreading from permissive to non-permissive cells.^87^ Based on these data collected since the onset of the pandemic, direct neuronal infection in humans may be plausible, but evidence suggests a greater likelihood of neuronal dysfunction arising from alternative, indirect pathways.

### Astrocytes

With placement between neuronal synapses and the cerebromicrovasculature, astrocytes provide a logical spatial relationship for intermediate signalling within the NVC response. However, many of the investigated signalling pathways postulated to link neuronal activity to increased cerebral blood flow (CBF) have been called into question. Evidence of intracellular Ca^2+^ increases in astrocytes with concomitant increase in microvascular diameter is achieved through activation of mGluR on astrocytes and inositol triphosphate (IP3) signalling, leading to uncaging of intracellular stores of Ca^2+^, activating phospholipase A_2_ (PLA_2_), and producing vasodilatory molecules [epoxyeicosatrienoic acids (EET) and prostanoids] that act on vascular SMCs and pericytes.^10,68^ Neuronal ATP binding to the astrocytic P2X receptor, which leads to an increase in intracellular Ca^2+^, has also been shown to affect vasodilation of cerebral micro-vessels, and inhibition of astrocytic P2X receptor suppressed the NVC response.^88^ Studies that challenge intracellular Ca^2+^ increases in astrocytes and their contribution to the vascular response have reported that the increases seen are too slow for the rapid coupling response and were shown to occur after vasodilation.^53,89^ The mGluR-IP_3_-PLA_2_ pathway has received criticism as well. In mice lacking IP_3_ receptors, functional hyperaemia occurred without preceding astrocytic intracellular Ca^2+^ increase,^90-92^ and NVC was unaffected in the absence of prostaglandin synthesis.^93-95^ Moreover, astrocytic potassium siphoning from neuronal excitatory transmission was proposed by Paulson and Newman^96^ as a plausible mechanism to elicit the vascular response. However, this has been largely refuted, showing that glial K^+^ siphoning does not contribute to the NVC response.^97,98^ While there is mixed evidence regarding glial contribution to the haemodynamic response, astrocytes remain an area of active research in neuro-(glial)-vascular coupling.

Aside from their contribution to NVC, astrocytes perform essential action within the brain parenchyma to ensure a stable, homeostatic microenvironment for proper neuronal function. Astrocytes function to regulate neurotransmitter release, buffer extracellular K^+^ following neuronal excitatory transmission, form the parenchymal side of the BBB, and modulate immune responses.^99^ However, during an insult (e.g. ischaemia, infection) they take on a ‘reactive’ phenotype where astrocytes hypertrophy, release pro-inflammatory signalling molecules and in cases of neuronal injury form a glial scar to isolate and contain the damage. This can be beneficial to prevent spread of injury and inflammatory processes to healthy neural tissues. During persistent activation, this can be detrimental to neuronal function, ensuing in loss of neural network connection, neuronal degeneration and impaired regeneration.^100^ Reactive astrocytes are a common finding in post-mortem examination and elevations in serum levels of glial fibrillary acidic protein (GFAP) and S100B were seen COVID-19 patients.^101,102^ Although evidence has suggested that astrocytes themselves are non-permissive to SARS-CoV-2 infection due to low or absent primary viral entry receptor ACE2, *in vitro* studies have revealed an alternative route for SARS-CoV-2 entry via the receptor basigin (BSG/CD147).^103^ Whether astrocytes become reactive to pro-inflammatory stimuli secreted from immune and/or endothelial cells, or if SARS-CoV-2 crosses the BBB and enters into astrocytes through alternative pathways, reactive astrocytic consequences on neuronal cell populations and network dynamics largely depends on vascular cell types with high permissibility.^104^

### Endothelia

The cerebromicrovascular endothelium is intimately related to the brain parenchymal cells. Direct interaction at the capillary level is made between astrocyte end feet and in small sections, neuronal synaptic bulbs. At the arteriole level, parenchymal vasculature is covered by SMC and astrocyte end feet, and penetrating arterioles are separated from the parenchyma by the perivascular space created by the glia limitans.^10^ Endothelial function within the peripheral vasculature is well known as a large contributor to vascular diameter and flow regulation. However, study of its role in the NVC response is relatively recent. Two predominant mechanisms are postulated to contribute to the vascular response of NVC, potassium inward rectifier (K_IR_) induced retrograde hyperpolarization signal propagation and local NO generation, both comprising endothelium-dependent models of NVC.^105,106^

Brain capillary endothelial cell K_IR_ channels are activated by extracellular K^+^ following neuronal excitation. Activation of these channels result in a propagated hyperpolarizing electrical signal upstream through connexins-based endothelial gap junctions and locally through myoendothelial gap junctions between pericytes and/or SMCs to elicit vasodilation by intracellular Ca^2+^ reduction.^105,107^ Endothelial NOS (eNOS) is postulated to be activated directly by neuronal activity and through the traditional neuronal-astrocytic signalling of NVC. NO is the most potent vasorelaxant in the brain, irrespective of NO generated by nNOS or eNOS, and blocking the signalling pathway of either enzyme results in a greater than 50% decrease in the NVC response *in vivo.*^108^ Within the neuronal-astrocytic mediated signalling framework, neuronal activity induces calcium wave propagation in astrocytes, elevates astrocytic ATP and hydrolyzed forms [i.e. adenosine diphosphate (ADP) and adenosine] binding to P2Y receptors on the cerebromicrovascular endothelium, contributing to eNOS mediated increases in NO and resulting NVC response.^53^ Moreover, selective inhibition of endothelial P2Y receptor and eNOS^−/−^ mice have a blunted NVC response, which provides evidence for the neuro-(glial)-vascular coupling hypothesis.^67^ Direct neuronal activation of endothelial NMDAR may also contribute to eNOS mediated dilation.^109^ Activity at NMDAR recruits eNOS through increased endothelial intracellular Ca^2+^ concentration. However, co-activation is required by astrocytic release of _D_-serine in response to neuronal activation,^110^ and future work is needed to determine how astrocytes promote _D_-serine release to favour endothelial NMDAR activation. Both models (i.e. retrograde hyperpolarizing signal and local eNOS mediated vasodilation) may work in tandem, as NMDARs are reported to be scarce in the capillaries but present in arterioles.^111,112^ Hence, at the capillary level extracellular increases in K^+^ concentration may initiate the start of the propagated vasodilatory response, with eNOS regulating upstream to increase precision of spatiotemporal CBF distribution, either through neuronal glutaminergic signalling or glial ATP release.

In contrast to neurons and astrocytes, the endothelium extensively expresses the ACE2 entry receptor for SARS-CoV-2. With the addition of common systemic comorbidities (i.e. T2D,^113^ hypertension^114^ and hypercholesterolaemia)^115^ that chronically disrupt vascular endothelial function, SARS-CoV-2 may be the tipping point to elicit multi-organ dysfunction, including brain pathologies contributing to cognitive and neuropsychiatric impairments. Indirect effects (i.e. leukocyte-driven cytokine storm mediated by neutrophils)^116^ are also a possibility in the pathogenesis of endothelial dysfunction. Both direct and indirect are likely to occur in tandem, as the SARS-CoV-2 virus has been seen within endothelial cells on histopathology reports^54^ and hypercytokinemia is known to cause deleterious effects on endothelial function.^117^

Following direct infection, S1 glycoprotein elicits elevated procoagulant and cytokine/chemokine markers^118,119^ and degradation of endothelial tight junctions making up the BBB.^120^ Moreover, the SARS-CoV-2 nucleocapsid protein (NP) can promote endothelial dysfunction, in contrast to NPs from other coronaviruses.^121^ The glycocalyx, a proteoglycan microstructure covering the endothelium essential for maintaining homeostatic function, is also damaged during SARS-CoV-2 infection.^122^ A healthy glycocalyx may prevent binding of S1 protein to endothelial ACE2; however, when damaged, the glycocalyx facilitates this interaction.^123,124^ Glycocalyx shedding is reported in both acute and recovered COVID-19 patients^125-127^ and glycocalyx treated with COVID-19 patient plasma triggered shedding and endothelial impairment,^122^ further exemplifying the persistent endothelial damage associated with COVID-19. Furthermore, endothelial infection by SARS-CoV-2 promotes endothelial cell injury and death,^54^ endothelial to mesenchymal transition,^128,129^ hyperpermeability,^130^ inflammation and elevations in leukocyte adhesion molecules,^119,130-135^ increased oxidative stress, decreased NO bioavailability,^65^ induction of endothelial senescence^136,137^ and impaired mitochondrial function.^138^ SARS-CoV-2-mediated mitochondrial dysfunction may also initiate a feedback-loop of excess mitochondrial ROS production and inflammation, which may contribute to the long-lasting endothelial dysfunction observed clinically.^65^

Clinically relevant cerebrovascular accidents, such as ischaemic and haemorrhagic stroke, are known complications of SARS-CoV-2. The risk of stroke from acute infection was proposed as a dependent consequence of COVID-19 severity.^139^ However, Veterans Affairs data showed post-COVID-19 patients (1 year after infection) have a significant increased risk of cardiovascular complications (including stroke) irrespective of acute severity of SARS-CoV-2 infection.^140^ Relative risk of stroke is 3–4 times higher in hospitalized COVID-19 patients compared to hospitalized patients without infection.^139^ Moreover, there is a 7–8 fold greater risk of stroke in patients with COVID-19 compared to patients with influenza.^141^ Cerebrovascular complications associated with COVID-19 are also more prevalent in patients with concomitant vascular risk factors.^139^ Aside from large vessel stroke, CSVD is a prevalent complication in both acute and recovered COVID-19 patients.^6^ CSVD is an often clinically silent disease affecting the small perforating arteries, arterioles, venules and capillaries.^37^ In our recently conducted systematic review of CSVD pathology in COVID-19, cerebral small vessel insults, both ischaemic lesions and microbleeds, were prevalent across studies, with microbleeds having a specific predilection to the corpus callosum, subcortical/deep white matter and juxtacortical white matter, differing from often comorbid conditions (i.e. acute respiratory distress syndrome, hypertension). Post-mortem studies also showed a high prevalence of subcortical microbleeds and increased burden of white matter hyperintensities (i.e. the most common marker of CSVD). In addition, histopathology reports from COVID-19 patients revealed CSVD pathology, including, endotheliitis, intravascular thrombosis, endothelial fibrosis and degeneration and BBB disruption.^6^ As the endothelium is the area of initial insult in large and small vessel stroke and CSVD pathology,^19^ this strengthens the ongoing consensus of COVID-19 as an endothelial disease.

### Mural cells

SMCs and pericytes represent the destination for neuro-(glial)-vascular signalling in arterioles and capillaries, respectively, to induce vasodilation and resulting increases in CBF to meet the nutritive and energetic needs of neurons. SMC relaxation in the brain is mediated by a wide variety of signalling pathways generated from neurons, astrocytes, and endothelium (e.g. NO, prostaglandin E2, EETs, etc.) as previously discussed. Intrinsic to the SMC, penetrating arterioles participate in regulation of flow, independent of signalling from the NVU (i.e. myogenic tone), by the mechanism of autoregulation.^142^ Exceeding the autoregulatory limit of SMCs disrupts NVU integrity by elevating downstream pressure in distal small vessels.^143^ At the transition zone between parenchymal arterioles and capillaries, there is a switch in mural cell predominance from SMCs to pericytes. Like SMCs, capillary pericytes are contractile, and participate in regulation of blood flow to help maintain relatively constant blood supply to the brain parenchyma.^144^ The vascular response to NVC (i.e. blood vessel dilation and resulting increased CBF) has been shown to occur first at the capillary level, approximately one second prior to arteriolar dilation in mice,^145^ indicating a role for pericytes as the initial ‘effectors’ of the vasodilatory response. As with SMCs, pericytes can respond to a wide variety of signalling molecules from neurons, astrocytes, and endothelia to initiate vasodilation at the most local level of the NVC response.^146,147^

Endothelial activation can cause detrimental effects on the brain parenchymal cells not only through dysregulation of blood flow, but also by secretion of pro-inflammatory mediators, ROS, and transmigration of leukocytes.^148,149^ SMCs, lining the media of arteries and arterioles in the brain, can also experience deleterious consequences following endothelial activation.^150,151^ Most relevant in the context of NVC is COVID-19-induced decrease in endothelium-derived NO bioavailability.^152^ As described, NO is a potent regulator of SMC vasorelaxation through activation of guanylyl cyclase mediated production of cyclic GMP (guanosine monophosphate). Hence, COVID-19-induced endothelial dysfunction may impair SMC regulation of vascular tone. Another consequence of SMC dysfunction mediated by endothelial activation is progression, or pre-existing history, of atherosclerosis. There is a bidirectional relationship between atherosclerosis and COVID-19, as presence of vascular comorbidities increases the severity of COVID-19 and COVID-19 itself can induce atherosclerotic-type vascular injuries.^153^ In response to endothelial dysfunction, SMCs proliferate and ingest cholesterol, contributing to early atherosclerotic lesions. Moreover, SMC-produced collagen deposits contribute to fibrous cap formation, potentially obstructing the lumen, leading to hypoperfusion. This is especially prevalent in the brain, as the majority of CSVD cases are driven by atherosclerosis progression.^154^ As COVID-19 has a strong association with small vessel pathologies,^6^ SARS-CoV-2-mediated endothelial dysfunction and its contribution to SMC proliferation and extracellular matrix deposits may elicit the atherosclerotic phenotype associated with CSVD. Atherosclerosis also affects the SMC intrinsic mechanism of cerebral autoregulation.^155^ Evidence suggests that autoregulation may be impaired in COVID-19 patients and could contribute to cognitive dysfunction.^156^ Aside from endothelium-mediated SMC dysfunction, disruption of endothelial barriers may allow for direct infection of SMC. However, the results indicate that SMCs and pericytes may not express the SARS-CoV-2 entry receptor (i.e. ACE2) and host protease (TMPRSS2) needed for viral fusion.^157^

Evidence indicates that COVID-19 may specifically be a microvascular endothelial disease, which leads to further exploration of SARS-CoV-2-induced effects on other microvascular cell types (i.e. pericytes). The susceptibility of pericytes to SARS-CoV-2 infection is conflicting. Whereas some report that pericytes are not susceptible to infection,^157^ multiplexed immunostaining of human brains revealed high ACE2 expression in pericytes.^158^ Further, CSF levels of platelet-derived growth factor β, a pericyte biomarker, were lower in COVID-19 patients, indicating a disruption in pericyte homeostasis.^158^ Moreover, an *ex vivo* study of hamster cortical brain slices indicated SARS-CoV-2 infection of brain pericytes, decreased functional ACE2 membrane activity and AngII-mediated capillary constriction,^159^ thus causing decreased cerebromicrovascular blood flow.^160^ However, the question of how SARS-CoV-2 crosses the BBB to reach the abluminal pericyte ACE2 receptor remains unanswered. Rather, we hypothesize that if SARS-CoV-2 has an effect on pericytes, it may be due to movement of S1 protein across the BBB via transcytosis^161^ or through BBB breakdown mediated by direct endothelial dysfunction and/or cytokine storm as a result of initial respiratory infection.^159,162^ Whether capillary pericyte or endothelium, concomitant ischaemia and microvascular dysfunction elicit elevations in pro-inflammatory cytokines, ROS and BBB disruption, which contribute to neuronal and astrocytic dysfunction mentioned above, and microglial reactivity mentioned below.^163-165^

### Microglia

Besides regulating immunological processes in the brain, microglia have also been linked to neurodegenerative diseases.^166,167^ While not until recently, evidence has suggested a role for microglia participation in the NVC response. Like astrocytes, microglia is well positioned between neurons and blood vessels and interact with both. Hence, critical appraisal of microglial role in the NVC response is an area of continued investigation. Mechanistically, microglia has direct purinergic connections between cellular components of the vasculature (i.e. endothelia, SMCs and pericytes). When purinergic microglial receptor (P2Y12R) was absent, vasodilation in response to CO_2_^168^ and functional hyperaemia in response to mouse whisker stimulation was diminished, suggesting microglial dynamic sensing of purines (e.g. release of neuronal ATP) may be critical for cerebromicrovascular vasodilatory response^169^ Moreover, microglia are a significant source of adenosine in the brain, potentially contributing to the NVC response through activation on purinergic receptors on the endothelium^67^ and/or modulation of neuronal transmission at synapses.^170^

In COVID-19 patients, post-mortem examinations reveal pronounced reactive microgliosis, with elevations in markers of phagocytosis.^81,82,171-173^ RNA-seq results have reported alterations of microglial transcriptome in COVID-19 patients compared to controls.^173^ Microglial differentially expressed genes in COVID-19 were related to inflammatory and cell death pathways.^81,173^ Hence, microglial activation is present in COVID-19 patients; however, how SARS-CoV-2 triggers this activation [e.g. direct infection, pattern recognition receptors (PRRs) endothelial mediated ROS/pro-inflammatory cytokines, etc.] is under investigation. Microglia respond to viral infection invasive to the parenchyma through PRRs, and elicit a pro-inflammatory response.^174^ Whether SARS-CoV-2 is neurotrophic is inconclusive, meaning that microglia activation may proceed through different pathways. Similar to pericytes, the S1 glycoprotein may cross the BBB and activate microglial PRRs. *In vitro* studies indicate that S1 glycoprotein induces elevations in pro-inflammatory cytokines produced by microglia.^175^ Danger-associated molecular patterns (DAMPs), such as extracellular matrix components and molecules released from damaged cells, may also activate microglia.^176^ In support of this postulation in COVID-19, studies of ischaemic stroke showed that BBB disruption and release of DAMPs rapidly activate microglia.^177^ As microglial activation is critical to respond to insults in the CNS (whether DAMPs or neurotrophic viruses), continued activation can prove to be detrimental to brain health.^178^ Little is known of the long-term complication regarding microglial activation following SARS-CoV-2 infection; however, some evidence does provide insight into the microglial response. Post-mortem analysis of cortical slices from COVID-19 patients has evidenced a transcriptomic profile of microglia similar to what is observed in neurodegenerative conditions.^81^ Preclinical models showed that S1 glycoprotein induced microglial activation associated with cognitive impairment.^179^ Also, a mild COVID-19 model resulted in microglial activation for up to seven weeks,^72^ thereby suggesting the possibility of microglial activation in the pathogenesis of long-term cognitive impairment. In addition, microglia may be directly infected if SARS-CoV-2 crosses the BBB, as evidenced by an *in vitro* study of the original, Delta and Omicron variants.^85^

## Neurovascular uncoupling in COVID-19

NVC impairment, or neurovascular uncoupling, is a prevalent mechanism in cognitive impairment^14^ and dementia.^53,180^ Vascular diseases are the predominant modifiable risk factors in the progression from normal aging to MCI, and from MCI to dementia. Moreover, SARS-CoV-2 is the newest ‘vascular risk factor’ that contributes to onset of cognitive impairment^28-30^ or worsening of an already pathologic cognitive decrement.^181^ While SARS-CoV-2's effects on the NVU have been recognized, its role in NVC responses has yet to be uncovered. In this section, we describe how acute SARS-CoV-2 infection and history of COVID-19 may disrupt NVC through the endothelium-dependent model. This focus is supported by the vast literature on COVID-19 as an endothelial disease and leads to increased translatability into the clinic for therapeutic intervention of long-standing, validated treatments known to restore endothelial health systemically.

While ROS are critical for homeostatic signalling pathways, persistent excessive levels are detrimental to normal cellular function^182^ and can damage tissues and organs such as the endothelium.^183^ Moreover, one of the most potent effects of elevated levels of ROS is uncoupling of eNOS [i.e. oxidation of co-factors (tetrahydrobiopterin)] and/or formation of reactive nitrogen species (i.e. peroxynitrite). NO plays an essential role in the control of vasomotor function throughout the body.^184^ In addition, this gasotransmitter is critical for maintaining an anti-inflammatory and anti-thrombotic state, and regulating platelet aggregation, SMC cell proliferation and migration, apoptosis, and endothelial barrier function.^185^ In the cerebromicrovasculature, the deleterious effects of ROS are evident, as endothelium-mediated NVC responses are impaired,^186-189^ and are associated with cognitive decline in preclinical models.^23,190^ In humans, modifiable vascular risk factors (e.g. obesity, hypertension, peripheral artery disease and type 2 diabetes), in which elevations in ROS and oxidative damage are central to their pathogenesis, impair endothelial function and NVC responses.^17,191,192^ In COVID-19, elevations in ROS are highly prevalent acutely and long-term,^193-195^ plausibly contributing to endothelial dysfunction as evidenced through mechanistic study of AT1R-mediated ROS generation. In support, preclinical models showed AngII-induced NVC impairment,^196,197^ even in the presence of endothelium-dependent vasodilators. Moreover, these findings are owed to increased AngII-induced cerebromicrovascular endothelial ROS production. Importantly, deleterious effects of AngII on NVC were attenuated by angiotensin receptor blockers (ARBs),^196^ and therapeutics that block signalling within the renin-angiotensin pathway showed beneficial effects on endothelial function in COVID-19 patients^54^ (discussed further in *Prevention and therapeutics in COVID-19-induced cognitive impairment).* Patients with acute COVID-19 had higher total oxidant status, oxidative stress biomarkers and lower anti-oxidant capacity than healthy controls.^193,194^ The same pattern was observed in convalescent COVID-19 patients.^195^ Oxidative stress and inflammation mutually enforce one another^117^ and contribute to the acute and convalescent COVID-19 thromboinflammatory phenotype.^198^ Pro-inflammatory binding to receptors on the endothelium elicits elevations in cytoplasmic and mitochondrial ROS, and in turn, production of cellular ROS activates transcription factors (e.g. nuclear factor κ B), which upregulate the synthesis of pro-inflammatory mediators and adhesion molecules. Moreover, this compromises vasoprotective sirtuin-mediated signalling pathways due to nicotinamide adenine dinucleotide (NAD^+^) depletion from pro-inflammatory PARP-1 [poly (ADP-ribose) polymerase 1] activation, further decreasing eNOS transcription, impairing the quiescent state of the endothelium.^117^ In sum, SARS-CoV-2 infection brings about acute and chronic endothelial activation,^54^ potentially mediating impairment of NVC in convalescent COVID-19 patients. While studies have yet to uncover the NVC response in acute and convalescent COVID-19 patients, recent work from Kuchler *et al*. showed that retinal microvascular reactivity in response to flickering light stimulation was decreased in COVID-19 patients compared to controls.^199^ This represents a crucial finding as the retina shares embryologic origin with the central nervous system^200^ and dilation in retinal micro-vessels is a direct consequence of NO release.^201^ Moreover, retinal microvascular reactivity is predictive of cognitive impairment.^202^ Additionally, there is a strong association with CSVD—both ischaemic and haemorrhagic—and neurovascular uncoupling.^203^ With COVID-19's predilection of CSVD pathology to subcortical regions,^6^ this highlights the need for a detailed, mechanistic study of NVC in vulnerable patient populations. Hence, early clinical evidence supports mechanistic study of microvascular impairment, plausibly contributing to impaired NVC as a mechanism for COVID-19-induced cognitive impairment.

## Prevention and therapeutics in COVID-19-induced cognitive impairment

**Figure 2:**
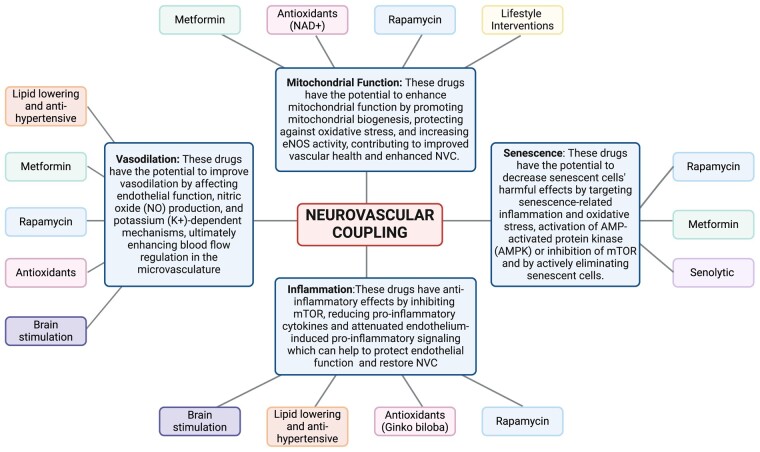
**Therapeutics targeting neurovascular uncoupling in COVID-19 induced cognitive impairment.** This schematic figure illustrates four key categories, each addressing specific aspects of neurovascular function affected by COVID-19: (i) Mitochondrial Function: Metformin, Anti-oxidants, Rapamycin and life-style interventions, such as time restricted eating; (ii) Senescence: Senolytics, Rapamycin and Metformin; (iii) Inflammation: Anti-oxidants Rapamycin, Statins, ACEI/ARBs and brain stimulation; (iv) Vasodilation Interventions: Anti-oxidants, Statins, ACEI/ARBs, brain stimulation, and metformin. While these interventions have shown promise in preclinical and clinical studies, further research is needed to fully elucidate their effects and potential benefits in the context of COVID-19-related cognitive decline. Created with BioRender.com.

Pharmacological therapy improves endothelial function in patients with history of COVD-19-induced long-term vascular dysfunction.^54^ In this section we present current evidence of therapeutics known to benefit endothelial function systemically in the context of COVID-19-induced NVC impairment. In addition, we highlight potential avenues for treatment options to be investigated in COVID-19-induced vascular dysfunction, ranging from life-style interventions to transcranial magnetic brain stimulation (Fig. 2). With this evidence, we hope to provide valuable insight into the mechanisms and therapeutic potential of these interventions to mitigate cognitive impairment following COVID-19. For detailed information on other therapeutic strategies targeting endothelial and cerebromicrovascular function not contemplated in this section, please refer to the excellent review by Xu *et al*.^54^

### Anti-oxidants

#### Vitamins C and E

Vitamins C and E both function as anti-oxidants through direct scavenging of free radicals, mitigating the detrimental consequences of cellular exacerbations of ROS.^204,205^ In addition, they have widely beneficial effects on the systemic vasculature, and at supraphysiological levels contribute to restoring endothelial function, partly through restoring eNOS function.^206-208^ As eNOS critically contributes to the NVC response, supplementation of these anti-oxidant vitamins may be useful to restore NVC and mitigate cognitive decrement. However, more studies are needed to determine their impact on the cerebromicrovascular endothelium-mediated response. In support of the beneficial effect of these vitamins on the brain, vitamin C supplementation has been associated with the reduction of free radicals, neuroinflammation, iron chelation, and amyloid-beta (Aβ) deposition.^209^ In vitamin C deficiency, preclinical models showed elevated oxidative stress markers and increased Aβ levels, emphasizing its role in mitigating neurodegenerative processes.^210^ Recent clinical trials have highlighted vitamin C's role in endothelial barrier integrity and redox homeostasis,^211^ providing further evidence of its potential as a therapeutic option in attenuating endothelium-dependent functional hyperaemic impairment. Moreover, plasma vitamin C concentrations were elevated in cognitively adept groups compared to individuals with cognitive impairment,^212^ highlighting the potential link between the known mechanistic aspects of vitamin C and cognitive function. Vitamin E, particularly in the form of α-tocopherol, has been proposed as a neuroprotective agent by demonstrating its ability to ameliorate learning and memory deficits, reduce oxidative stress, diminish Aβ deposits, and enhance cognition within Alzheimer’s disease models.^213,214^ Moreover, clinical trials show that 800–2000 IU/day of vitamin E to Alzheimer’s disease patients exhibited decreased functional decline compared to placebo, and oxidative stress was attenuated.^213,215^ Hence, both vitamin C and E may be beneficial in restoring NVC responses and cognition in acute and convalescent COVID-19 patients with cognitive impairment.

#### Ginkgo biloba

Ginkgo biloba special extract EGb 761^®^ emerges as a promising contender for addressing NVC and mitigating COVID-19-induced cognitive impairment. EGb 761^®^ has been proposed as a potential remedy to alleviate cognitive symptoms persisting beyond three months post-COVID-19 infection.^216,217^ This extract has demonstrated a multifaceted approach to combatting the complications arising from SARS-CoV-2 infection, particularly by targeting oxidative stress and inflammation, both integral to the pathogenesis of post-acute sequelae.^218^ Its active constituents, including flavonoids, biflavones, terpene trilactones and ginkgolic acids, contribute to its antiviral potential and ability to modulate various stages of the coronavirus life cycle.^219^ The neuroprotective properties of EGb 761^®^ were elucidated through studies involving cerebral injury induced by ischaemia/reperfusion (I/R) in rats. These investigations demonstrated reductions in malondialdehyde (MDA) levels, pro-inflammatory cytokines (TNF-α and IL-1β) downregulation, increased expression of anti-inflammatory cytokines (IL-10) and enhanced enzymatic anti-oxidant activities. Notably, EGb 761's beneficial effects on I/R injury are attributed to its ability to inhibit inflammation, subsequently reducing oxidative stress.^219^ These mechanisms align with the potential to mitigate cognitive deficits experienced post-COVID-19 infection. EGb 761’s efficacy in treating cognitive impairment, coupled with its anti-inflammatory effects and enhancement of neuroplasticity, makes it a potential low-risk treatment option for patients experiencing persistent cognitive symptoms following COVID-19 infection.

#### Resveratrol

Resveratrol, a natural polyphenol abundant in red grapes and red wine, has established anti-oxidant, anti-inflammatory, and anti-tumorigenic properties.^220^ Increasing evidence suggests that redox balance plays a pivotal role in viral infections, with viruses capable of depleting glutathione levels, and interventions targeting oxidative stress showing potential.^221,222^ In the context of COVID-19, resveratrol has demonstrated antiviral activity against SARS-CoV-2 through inhibition of viral replication, while reducing cytotoxicity.^223,224^ Notably, resveratrol has been shown to selectively inhibit key proteases of SARS-CoV-2 in vitro.^225^ Clinical trials have also provided insights into resveratrol's potential in combating COVID-19-related complications. A randomized, double-blind, placebo-controlled proof-of-concept trial revealed that resveratrol supplementation was associated with a lower incidence of hospitalization, COVID-related emergency room visits, and pneumonia among mild COVID-19 outpatients.^226^ Resveratrol's impact on the renin-angiotensin system (RAS) and ACE-2 expression positions it as a candidate for attenuating SARS-CoV-2 entry and viral shedding.^227^ Resveratrol treatment in elderly mice restored NVC and acetylcholine-induced responses. This effect was linked to a decrease in NADPH (nicotinamide adenine dinucleotide phosphate) oxidase expression and reduced oxidative/nitrative stress markers in the cortex. Additionally, resveratrol mitigated age-related increases in ROS production in cultured cerebromicrovascular endothelial cells. This suggests that resveratrol's impact on cortical NVC in aged mice is likely due to the restoration of cerebromicrovascular endothelial function through downregulation of NADPH oxidase-derived ROS production. These cerebromicrovascular benefits of resveratrol could contribute to its protective role in preserving cognitive function during aging.^228^ Human studies and clinical trials have also explored resveratrol's effects on cognitive function. In a randomized trial, acute resveratrol administration increased CBF in a dose-dependent manner, though cognitive improvements were not immediate.^229^ Chronic resveratrol supplementation demonstrated improvements in cognitive function, specifically accuracy and reduction of fatigue.^230^ The benefits of resveratrol on cognitive performance were observed in overweight older adults,^231^ where memory retention and functional connectivity between the hippocampus and other brain areas improved. Similar effects were reported in patients with T2D, indicating potential cognitive benefits through enhanced cerebrovascular function.^232^ These findings collectively underscore the potential of resveratrol as a therapeutic option to prevent and treat COVID-19-induced cognitive impairment and emphasize its role in NVC.

#### Cocoa

Cocoa, in its natural unprocessed form, contains abundant catechins and epicatechins, which are flavanols belonging to the subclass of flavonoids.^233^ Cocoa intake holds the potential to mitigate cognitive decline by promoting improved cerebral vasodilation, blood flow, perfusion, and angiogenesis.^234-236^ Notably, epicatechin possesses the ability to be readily absorbed, cross the blood–brain barrier, and exert physiological effects in the brain.^236^ Studies showed that flavonoids, particularly in higher amounts (500–750 mg/day), have the potential to enhance memory and executive function in healthy older adults.^237^ Clinical trials have been conducted to explore the cognitive effects of cocoa consumption. One such trial, Cocoa Supplement and Multivitamin Outcomes Study for the Mind, assessed the impact of daily cocoa extract administration (containing 500 mg flavanols) over a period of 3 years in older adults. The results indicated no significant cognitive benefit from the cocoa extract. In contrast, another trial involving older participants revealed that 30 days of cocoa consumption^238^ led to increased NVC and improved cognitive performance, particularly in individuals with impaired NVC at baseline.^239^ While the potential of cocoa in vascular health and cognitive enhancement is evident, further research are needed to better understand its role in preventing and treating COVID-19-induced cognitive impairment.

#### Nicotinamide adenine dinucleotide

Nicotinamide adenine dinucleotide has numerous roles in maintenance of cellular functional and metabolic homeostasis. Importantly, NAD^+^ is a rate-limiting substrate for sirtuin activity, which is critical for maintaining proper mitochondrial function (i.e. mitophagy and biogenesis). Activation of sirtuin 1 protects the cell from excessive ROS production^240,241^ and supplementation of sirtuin 1 activator SS-31 enhanced cerebromicrovascular endothelial function, functional hyperaemia, and cognitive performance. Moreover, NAD^+^ and/or precursor supplementation reduced cerebromicrovascular endothelial cell mitochondrial ROS production, improving NVC and cognition in a sirtuin-dependent manner.^242^ NAD^+^ bioavailability decreases with age and during viral infection, in part due to PARP1 consuming enzymes that interact with, and perpetuate pro-inflammatory and oxidative signalling.^117,243^ In addition, reduced NAD^+^ levels play a critical role in neurodegenerative disorders and vascular dementia. Preclinical models have evidenced that with supplementation of NAD^+^, cognitive impairment was significantly ameliorated.^244^ In light of low NAD^+^ levels association with poor outcomes in COVID-19 patients,^243,245^ NAD^+^ depletion may contribute to impairment of NVC, and cognition seen in preclinical models. Clinical trials are needed to assess the effects of NAD^+^ supplementation on NVC in convalescent COVID-19 patients.

#### Alpha-ketoglutarate

As an intermediate of the Krebs cycle, alpha-ketoglutarate (AKG) plays essential roles in metabolism and metabolic processes within the human body. Importantly, in the context of therapeutic treatment in COVID-19, AKG functions as an anti-oxidant, potentially alleviating the detrimental consequences of elevated oxidative stress,^246^ AKG's contribution to brain health is multifaceted. AKG deamination forms glutamate and can be further decarboxylated to GABA (gammaaminobutyric acid), the two main excitatory and inhibitory neurotransmitters in the brain, respectively. Moreover, AKG alleviates oxidative damage in the vasculature, reducing stiffness, and increasing NO production.^247^ In COVID-19, AKG supplementation inhibited SARS-CoV-2 replication, reduced inflammation and thrombosis, rescued lung function, and restored oxygen saturation in SARS-CoV-2 infected animals.^248^ Hence, with the benefits of enhanced neuronal function, neurotransmitter regulation, and vascular homeostasis, AKG positions itself as a prime supplement to alleviate NVC disruption and cognitive impairment in COVID-19.

### Life-style and non-invasive interventions

#### Time-restricted eating

Intermittent fasting is a dietary pattern of eating that involves extended periods (typically 16–48 hours) of reduced or no food consumption, followed by regular eating intervals.^249^ Animal models’ studies showed that intermittent fasting can activate adaptive cellular stress signalling pathways, enhance mitochondrial metabolism, promote DNA repair, improve endothelial function and stimulate autophagy, resulting in benefits for chronic diseases including diabetes, cardiovascular disorders, cancer and neurological conditions like Alzheimer’s disease.^250,251^ As such, through these beneficial actions on endothelial cells it is highly plausible that NVC can be rescued through varying time-restricted eating paradigms. By leveraging these adaptive mechanisms, intermittent fasting enhances fat oxidation as an energy source, lowers serum glucose levels and preserves spatial memory function.^252,253^ Intermittent fasting holds promising potential for enhancing vascular health and addressing COVID-19-induced cognitive impairment. The connection between metabolic brain studies using FDG-PET (fluorodeoxyglucose-positron emission tomography) imaging and cognitive deterioration in post-COVID-19 patients underscores the importance of addressing cerebral glucose hypometabolism, a hallmark of conditions like MCI and Alzheimer’s disease.^254-259^ This hypometabolism can be mitigated through dietary interventions that elevate serum ketone bodies, such as intermittent fasting.^260-262^ In a study evaluating the outcomes of patients engaging in periodic fasting prior to COVID-19 diagnosis, a lower risk of hospitalization or mortality was observed among those practicing periodic fasting compared to non-fasters.^263^ Hence, further research and clinical trials are needed to elucidate whether intermittent fasting can prevent and treat COVID-19-induced cognitive impairment.

#### Transcranial direct current stimulation

Transcranial direct current stimulation (tDCS) is a non-invasive brain stimulation technique that modulates brain vascular function, enhances synaptic plasticity and reduces pro-inflammatory cytokines,^264^ making it a viable candidate for treating COVID-19-induced cognitive dysfunction through NVC-mediated mechanism.^265^ Prior research has demonstrated the effectiveness of tDCS in non-COVID-19 samples and in combination with cognitive tasks for neurorehabilitation and cognitive enhancement.^266,267^ A pilot case series involving four patients with long-COVID cognitive symptoms showed promising trends. The patients underwent 20 daily sessions of bilateral prefrontal tDCS combined with online cognitive training. Although this pilot study lacked statistical power, it demonstrated several encouraging outcomes: improvement in depression symptoms, reduced self-reported cognitive and emotional symptoms, enhanced processing speed and executive functioning, and better delayed and immediate recall.^268^ In another case report, two patients demonstrated remarkable enhancements in physical, cognitive, emotional, and functional aspects, allowing them to successfully return to the workforce and resume their previous activities. In this case, tDCS was also delivered over left dorsolateral prefrontal cortex and combined with physical exercise, online adaptive computerized cognitive training and guided mindfulness meditation.^269^ However, preliminary results from an ongoing study of tDCS on cognitive impairment in post-COVID patients indicated that there was no significant difference between the active tDCS and sham groups in terms of cognitive test results after four weeks.^270^ Overall, tDCS emerges as a promising intervention where multiple ongoing studies are dedicated to unravelling the impact of tDCS on cognitive dysfunction subsequent to COVID-19 (NCT04944147; NCT05389592, NCT05965739). These studies exemplify the ongoing efforts to delve into tDCS's potential within the context of post-COVID-19.

#### Transcranial magnetic stimulation

Transcranial magnetic stimulation (TMS) is a non-invasive technique capable of directly modulating neuronal activity and synaptic plasticity within the cortex and interconnected brain regions.^271^ This modulation extends to a range of neural processes, encompassing NMDAR activity, GABA mediated inhibition, and the activation of neuromodulators like acetylcholine, dopamine, norepinephrine, and serotonin.^272-274^ Moreover, repetitive TMS also influences nonneuronal processes, including alterations in blood flow.^273,275^ While its impact on endothelial function is not extensively studied, TMS significantly contributes to the exploration of cognitive mechanisms and behavioural plasticity within the human brain. Additionally, TMS has demonstrated effectiveness in ameliorating cognitive deficits among patients with MCI and Alzheimer’s disease.^276^ In a recent case series involving 23 patients with Long-COVID, TMS mitigated subjective and objective depressive symptoms, as well as improvements in cognitive impairments such as brain fog, were observed.^277^ Moreover, executive function, as assessed by the Trail Making Test, showed notable improvement following TMS treatment. A recent case study, utilizing personalized TMS guided by EEG, reported a positive outcome in a 36-year-old patient experiencing lingering COVID-19-induced “brain fog”. The treatment yielded improvements in mood, olfactory function, and cognitive clarity.^278^ These findings highlight TMS's potential as a therapeutic path to tackle COVID-19-induced cognitive impairment. Nevertheless, further investigations are warranted to explore into the full scope of TMS's effectiveness in ameliorating cognitive deficits arising from COVID-19, as well as to explore whether these effects are contingent upon NVC.

### Prebiotics, probiotics, fecal microbiota transplant

Microbial gut composition is strongly associated with clinical cognitive outcomes,^279^ and dysbiosis of the gut microbiome may contribute to neurocognitive disorders, such as Alzheimer’s disease.^280^ Gut dysbiosis has been described as a mechanism of long-COVID pathogenesis due to the direct and indirect links between SARS-CoV-2-induced NVU and intestinal barrier impairment that disrupts the gut-brain axis.^281^ A randomized, double-blinded, placebo-controlled, multicenter clinical trial determined that probiotics promoted increases in cognitive performance and elevations in serum brain-derived neurotrophic factor (BDNF) level in older adults.^282^ Additionally, preclinical models have shown that prebiotics may also provide promise in alleviating cognitive impairment through modulation of neurotransmitters, BDNF, and NMDAR subunits.^283^ Fecal microbiota transplantation (FMT) is a procedure to introduce a healthy donor fecal matter into the gastrointestinal tract of a patient. Recent evidence has shown that introduction of a healthy microbiome to patients with cognitive impairment and *Clostridioides difficile* infection improved cognition compared to the control group with antibiotic treatment.^284^ Moreover, FMT has been proposed to have a positive impact on the treatment of Alzheimer’s disease through mechanistic pathways involving anti-inflammation and synaptic plasticity.^285^ These therapies may have a positive influence on COVID-19 induced NVU disruption due to their promising neuromodulatory effects.

### Lipid lowering and anti-hypertensive pharmaceuticals

Statins are first-line therapy for individuals with hypercholesterolaemia and atherosclerotic artery disease. These drugs have potent effects on the vasculature with lipid lowering, anti-inflammatory, anti-oxidant, and anti-thrombotic properties^54^ Hence, statin therapy may provide great benefit to the cerebrovasculature, and in turn, rescue endothelium-dependent vasodilatory regulation. While statins are known to increase the expression of ACE2, clinical data supports the use of statins in COVID-19 patients.^286^ Statins have been shown to lower the risk of mortality in COVID-19 patients^287^ and mechanistically, statins attenuated endothelium-induced inflammation and pro-inflammatory signalling.^54^ Statins also showed benefit in preclinical Alzheimer’s disease models, completely rescuing cerebrovascular reactivity, endothelial NO production, and NVC responses.^288^ Moreover, statins have improved cognitive function,^289^ exemplifying long-standing statin therapy as a potential mediator to attenuate cognitive decrement following SARS-CoV-2 infection. Like statins, angiotensin converting enzyme inhibitors (ACEI) and AngII receptor blockers (ARBs), were initially thought to increase vulnerability to SARS-CoV-2 infection due to their role in upregulation of ACE2. However, discontinuation of ACEI/ARBs has been associated with poor outcomes in COVID-19 patients.^54^ Hence, professionals from cardiovascular societies suggest continuation of ACEI/ARBs during COVID-19 infection.^290^ A beneficial effect of these lipid lowering and anti-hypertensive drugs could be their action in upregulation of ACE2, as virus-treated microvascular endothelial cells exposed to an ACE2 agonist reversed SARS-CoV-2 induced hyperpermeability, stabilizing endothelial barrier integrity.^54,291^ In addition, ARBs showed a positive effect on the NVU by rescuing NVC response through restoration of cerebral endothelial cell NO bioavailability in preclinical models,^292^ further strengthening the premise of lipid lowering and anti-hypertensives as treatment for NVC impairment in COVID-19.

### Senolytics

Increases in cellular damage and oxidative stress can trigger cellular senescence, a complex stress response^293^ and potentially underlying pathophysiologic mechanism contributing to decreased cognitive function in cognitive impairment and dementia.^294^ In response to this stress, the endothelium can switch to a senescent phenotype, which contributes to loss of vascular tone regulation, and impairment of anti-coagulant, anti-oxidant, and anti-inflammatory quiescent properties, in part, by impairing NO production.^295^ Senolytic drugs have shown promise in preclinical models to restore the NVC response and hippocampus dependent learning and memory.^293^ In a preclinical chemotherapy model, paclitaxel induced endothelial senescence contributed to a decreased NVC response and cognitive impairment. When senescent endothelial cells were eliminated, microvascular density was increased, NVC was restored, and cognitive performance improved.^296^ Moreover, a senescent endothelial phenotype secretes pro-inflammatory molecules and contributes to oxidative damage, capable of disrupting the BBB and inducing neuroinflammation, which is associated with impaired NVC.^296^ Elimination of senescent endothelial cells in a preclinical model of whole brain irradiation restored BBB integrity and increased cortical capillarization.^297^ Clinically, virally induced senescence markers have been detected in lung endothelial cells^298^ and there is preclinical evidence of senolytic treatment reducing mortality rate in SARS-CoV-2 infected mice.^299^ Hence, the molecular and physiological consequences of elevations in pro-inflammatory cytokines and ROS may be due to phenotypic change of endothelial cells. Senolytic clinical trial to modulate the progression of Alzheimer’s disease (SToMP-Alzheimer’s disease) showed decreases in senescence associated secretory phenotype factors; however, cognition and neuroimaging endpoints showed no difference compared to baseline.^300^ Further clinical trials are needed to assess the effectiveness of senolytics, and how these drugs can potentially be used to mitigate cognitive deficits in COVID-19 patients.

### Metformin

Metformin is a low cost and common diabetes drug worldwide. Aside from its beneficial effects of lowering blood glucose and increasing insulin sensitivity, metformin exhibits potent anti-inflammatory and antioxidative properties. Metformin showed benefit in decreasing endothelial oxidative stress and preserving eNOS phosphorylation in high fat diet fed rats.^301^ Preclinically, metformin also showed cognitive benefits and restoration of cerebrovascular function.^302^ In addition, clinical results conclude that metformin significantly reduces cardiovascular complications^303^ and a recent meta-analysis of 10 studies suggests that metformin reduces impairment of cognition in patients with T2D.^304^ In the context of COVID-19, metformin outpatient treatment reduced the incidence of Long-COVID by 41%.^305^ Metformin is a safe and globally available pharmacologic, and due to its favourable outcomes in COVID-19 and positive effects on the NVU and cognition, this drug may prove useful for restoration of the NVC response and cognitive function.

### Rapamycin

Rapamycin is a macrolide antibiotic that exhibits extensive immunosuppressive activity, with clinical uses that span from preventing organ transplant rejection to cancer.^306^ Mechanistically, rapamycin inhibits the molecular target of rapamycin (mTOR), which plays a key role in viral replication and production of pro-inflammatory cytokines.^307^ Since 2009 when Harrison et. al. discovered rapamycin's use in extending life span,^308^ rapamycin has been at the forefront for treatment of age-related conditions, including cardiovascular and neurodegenerative diseases.^306^ Due to rapamycin's inhibitory effects on the mTOR pathways and potential as an anti-aging drug, rapamycin is currently being investigated in clinical trials to mitigate COVID-19-induced detrimental cellular and molecular consequences across organ systems.^307^ Importantly, rapamycin shows preclinical evidence of NVC and memory rescue in a preclinical Alzheimer’s disease model.^309^ Studies examining the effect of rapamycin in the rescue of NVC response revealed that inhibition of mTOR restored cerebrovascular function by rescuing eNOS function.^310,311^ In addition, rapamycin improved nNOS activation, all-in-all, indicating that the mTOR pathway has critical regulation on the most potent stimuli of the NVC response, NO.^309^ As we await clinical trial results of rapamycin efficacy for prevention of post-COVID-19 effects, mTOR inhibition seems to be a promising avenue to restore cerebromicrovascular and cognitive function in COVID-19 patients.

## Conclusion

Over the past 3 years, the detrimental consequences of acute and long-term COVID-19 have been evident, with systemic spread of endothelial impairment as a major driver of brain pathologies. SARS-CoV-2-induced NVU impairment has perpetuated a cognitive sequela whereas of now, we lack the knowledge regarding the specific time point at which convalescence to acute infection will render individuals unsusceptible. In this review, we presented evidence of our hypothesis that long-term cognitive impairment is due to a compromise of the endothelium-mediated mechanism of NVC. Moreover, there is therapeutic potential of many low-risk treatments, non-invasive procedures, and life-style changes, which may provide benefit in COVID-19-induced cognitive impairment by rescuing the NVC response. Paths forward to achieve the goal of nullifying cognitive impairment associated with COVID-19 will take a combined effort from bench to bedside research, investigating mechanisms and pharmacological trials to restore cerebromicrovascular health and in turn, reconstitute cognitive processes.

## Data Availability

Data sharing is not applicable to this article as no new data were created or analyzed in this study.
